# Heterosexual men who purchase sex and attended an STI clinic in Israel: characteristics and sexual behavior

**DOI:** 10.1186/s13584-018-0213-4

**Published:** 2018-06-27

**Authors:** Rivka Rich, Alex Leventhal, Rivka Sheffer, Zohar Mor

**Affiliations:** 1 School of Public Health, Hebrew University, Jerusalem, Israel; 2 Ministry of Health, Public Health Services, Jerusalem, Israel; 3 Tel Aviv Department of Health, Tel Aviv, Israel; 4 School of Public Health, Tel Aviv University, Tel Aviv, Israel

**Keywords:** Sexual behavior, Sex-workers, Heterosexual behavior, Sex-purchasing

## Abstract

**Background:**

Commercial sex shares a role in HIV and sexually transmitted infections (STI) transmission. Men who pay for sex (MPS) may transmit HIV/STI to other populations which are low-risk. This study aimed to test our hypothesis that MPS engage in high-risk sexual behaviors associated with HIV/STI transmission more so than non-MPS.

**Methods:**

This cross-sectional study included heterosexual men who attended an STI clinic between 2003 and 2010. Demographic, clinical, behavioral and laboratory data were compared between MPS and non-MPS to identify factors associated with high-risk sexual behavior and STI-burden.

**Results:**

Of the first visits of 6156 heterosexual men who attended the STI-clinic during the study period, 1649 (26.7%) were MPS. MPS were more commonly older, married and non-Israeli born compared with non-MPS. MPS were more likely to engage in risk-behaviors associated with HIV/STI-transmission, including a greater number of lifetime sexual partners, substance use and previous STI diagnoses.

Determinants associated with STI-diagnoses at the current visit included being non-Israeli born, presenting with STI symptoms, reporting a greater number of lifetime sexual partners and having sexual encounters with non-Israeli individuals.

**Conclusions:**

Approximately 25% of all men who attended the clinic were MPS. They were more likely to engage in risk-behaviors associated with HIV/STI transmission compared to non-MPS. These findings highlight the need to establish interventions for MPS that both continue to encourage condom use and address the potential perils pertaining to risky sexual behaviors.

## Background

Societal opinion regarding female commercial sex workers (FCSW) is diverse and has changed over recent decades. While some countries allow regulated prostitution (such as Nevada in the United States and the Netherlands), other have banned paid sex. Nordic countries have employed a different model where it is illegal to pay for sex, but not to sell sex i.e., the client is criminalized, but not the FCSW [1]. In Sweden, where this model was first applied, a reduction in street prostitution was observed without causing a rise in indoor prostitution (where it occurs in more clandestine settings) [2]. Prostitution in Israel is legal yet not regulated, but procuring is illegal. Recently, a new law reform has recently received preliminary approval by the Israeli Parliament’s ministerial committee and will be brought to the parliament for the final legislative approval [3], constituting similar regulations as the Scandinavian model. In light of this legal shift, this study focuses on men who pay for sex (MPS), as they are key players, driving the demand for commercial sex.

Unlike widely studied public health aspects of FCSW [4, 5], only limited studies have concentrated on MPS, as they are usually a hidden population that is unlikely to participate in structured studies [6, 7]. Representative samples of the general population in European countries have shown the percentage of men who have ever purchased sex in the past year varies greatly, ranging from 1%–11% [6]. Studies have also shown that MPS report riskier sexual behavior than non-MPS, and are considered to be at a high risk for acquiring HIV and sexually transmitted infections (STI) [7–9]. Furthermore, they can act as agents for transmitting HIV/STI both to FCSW and to low-risk groups in the community, while performing unprotected sex with their steady partners or casual non-paid sexual encounters, a phenomenon termed “bridging” [7, 10].

According to an Israeli National survey performed in 2014, it was estimated that there are 10,463 FCSW and 525 male commercial sex workers in Israel, with the majority concentrated in the Tel Aviv metropolitan area [11]. In a representative survey of Jewish males aged 18–44, approximately 20% of the heterosexual men reported ever-purchasing sex [12].

This study is the first to compare demographic attributes, behavioral characteristics and STI burden in Israel between heterosexual MPS and non-MPS who attended an STI clinic in Israel. The Levinski STI clinic is a walk-in community service clinic, operated by the Tel Aviv department of health, that provides counseling, testing and treatment anonymously and free of charge [13]. Its services are accessible to citizens and non-citizens alike. Males who attend this clinic do so as they may prefer not to disclose to their personal doctors sensitive issues such as risky sexual behaviors, including paying for sex. We hypothesized that MPS will demonstrate greater risky sexual behavior than non-MPS.

## Methods

This cross-sectional study included data retrieved from the individual medical files of all heterosexual men who attended the STI clinic between 2003 and 2010.

To comply with the cross-sectional study design, only the first clinic visit was included. The data was collected from a computer-assisted structured medical interviews performed by the clinic’s medical staff. Questions included the reason for their visit (clinical symptoms or routine screening), age of first sexual debut, number of previous sex partners, the gender of their partners (men, women or both), previous STI diagnoses, sexual practices, past encounters with FCSW, as well as condom and substance use. Medical examination by a trained physician and STI tests were performed according to the patient’s risk behavior and the clinical symptoms (urethral secretion, burning, or bleeding; sore, scratch or penile skin changes; chest/palms/soles skin rash) presented at the visit. The final analysis only included heterosexual men between the ages of 18 and 80 years that had a complete medical file.

Laboratory tests included serology tests of blood samples for HIV, hepatitis B surface antigen (HBsAg), VDRL (Venereal Disease Research Laboratory), TPHA (Treponema pallidum hemagglutination) and FTA (fluorescent treponemal antibody blood). Pharyngeal and urethral swabs for *N. Gonorrhea* were detected by Gram staining and culture. Urine was collected for nucleic acid amplification tests (NAAT) to detect *N. Gonorrhea* and *C. trachomatis*. Men who presented positive serology to HIV, HBsAg or infectious syphilis (VDRL titers higher than 1:8 with a positive FTA of TPHA), pharyngeal or urethral *N. Gonorrhea*, urethral *C. Trachomatis* during their current visit were classified as having an STI. Positivity rates were calculated as follows- the number of positive results was divided by the number of tests performed for each of the pathogens.

Risk factors associated with HIV/STI transmission in this current study included infrequent use of condoms during vaginal sex (ranging from never used to sometimes using a condom), performing sex under the influence of drugs and multiple sex partners [12, 14]. Multiple sex partners was defined as greater than the median number of lifetime sexual partners of the entire study population.

MPS was defined as men who reported one or more occasions of purchasing sex. A trend analysis of men who paid for sex during the study period was performed using the Chi-square test to yield the linear-by-linear association test. Demographic, behavioral and laboratory data were compared between MPS and non-MPS and also between men who had one or more STI diagnosis to men who were STI-free. The demographic and behavioral characteristics of MPS was also analyzed according to the frequency of purchasing sex (once, rarely and often). The Chi-square test was used to compare between categorical variables while the student’s *t*-test was used to compare continuous variables. A *P*-value of < 0.05 was considered statistically significant. Logistical regression included attributes which were statistically significant in the univariate analyses after testing for co-linearity with other independent variables, and was used to identify variables significantly associated with purchasing sex and having at least one STI diagnosis at the current clinic visit (SPSS, v-21). The results of the analysis are presented as odds ratio (OR) and 95% confidence interval (CI).

## Results

The first visits of 6719 heterosexual who attended the STI-clinic between 2003 and 2010 were recorded, while 6156 (91.6%) met the inclusion criteria and were included in the final analysis. The average age of the study population was 32.3 ± 9.9 years (range: 18–80, median of 29), and the majority was single (*N* = 4360, 82.5%) and Israeli born (*N* = 4211, 69.5%).

**Fig. 1:**
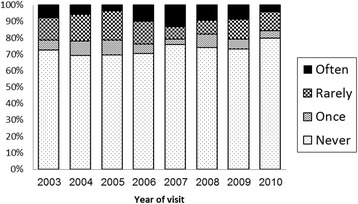
Trend analysis of men who paid for sex during the study period

Of the participants, 1649 (26.7%) reported that they purchased sex at least once in the past. A trend analysis indicated that the percentage of men purchasing sex varied over the years ranging between 20.1–30.6%, *p* < 0.001 (Fig. 1).

MPS were more commonly older, married and non-Israeli-born (mostly from the Former Soviet Union [FSU], Asia and Africa) compared to the non-MPS (Table 1). According to the univariate analysis, MPS were also more likely to present STI related symptoms at the visit, report a greater variety of sexual practices, more likely to perform sex with non-Israeli born partners within the three months prior to the clinic visit, used drugs during sex and reported previous STI diagnoses compared to non-MPS. Determinants associated with sex purchasing in the multivariate analysis included older age, being married, born in the FSU or Asia, presenting with STI symptoms, a greater number of lifetime sexual partners, performing sex under the influence of drugs and reporting a previous STI diagnosis (Table 1).

**Table 1:** Characteristics of heterosexual men visiting the STI clinic who purchased sex vs. those who did not purchase sex

		Univariate analysis				Multivariate analysis	
		Paid for sex 1649 (%)	Did not pay for sex 4507 (%)	OR (95% CI)	*P*	OR (95% CI)	*P*
Age	18–29	681 (41.5)	2426 (54.0)	1 (ref)	< 0.001	1 (ref)	0.005
	≥30	964 (58.5%)	2060 (46.0)	1.6 (1.4–1.8)		1.4 (1.1–1.9)	
Relationship status^a^	Single (single, divorced & widowed)	1014 (75.5)	3346 (85.0)	1 (ref)	< 0.001	1 (ref)	0.03
	Married	331 (24.5)	582 (15.0)	1.4 (1.2–1.6)		1.46 (1.02–2.0)	
Country of birth	Israel	1083 (66.5)	3128 (70.5)	1 (ref)		1 (ref)	
	Western Countries	48 (3.0)	176 (4.0)	0.7 (0.5–1.0)	0.1	0.7 (0.3–1.5)	0.4
	North Africa and Middle East	40 (2.5)	36 (1.0)	3.2 (2.0–5.0)	< 0.001	2.0 (0.7–5.6)	0.2
	The FSU	328 (20.0)	648 (14.5)	1.4 (1.2–1.6)	< 0.001	1.6 (1.1–2.3)	0.01
	Asia	38 (2.5)	54 (1.2)	2.0 (1.3–3.0)	< 0.001	2.7 (1.02–7.6)	0.03
	Africa	72 (4.5)	358 (8.0)	0.5 (0.4–0.7)	< 0.001	1.0 (0.6–1.6)	0.7
	South America	17 (1.0)	44 (1.0)	1.1 (0.6–1.9)	0.7	1.1 (0.3–3.8)	0.8
Reason for attending clinic	Symptomatic or sex with a symptomatic partner	378 (23.0)	800 (18.0)	1.3 (1.2–1.5)	< 0.001	1.8 (1.3–2.4)	< 0.001
	Self-referral screening	1271 (77.0)	3707 (82.0)	1 (ref)		1 (ref)	
Age sexual debut < 18 years		874 (56.0)	2185 (50.5)	1.2 (1.1–1.4)	< 0.001	1.0 (0.8–1.3)	0.8
≥ 11 sexual partners during lifetime^a^		1134 (72.0)	2322 (53.0)	2.0 (1.8–2.3)	< 0.001	1.6 (1.2–2.1)	< 0.001
≥6 sexual partners within the last 3 months		303 (19.0)	391 (9.0)	2.3 (1.9–2.7)	< 0.001		
Irregular or no use of condom during vaginal sex		1234 (79.0)	3447 (82.0)	0.8 (0.7–0.9)	0.03	0.9 (0.7–1.4)	0.8
Irregular or no use of condom during anal sex		430 (77.5)	977 (78.5)	0.9 (0.7–1.2)	0.6		
Performed sex with a non-Israeli partner within the last 3 months		126 (28.5)	315 (21.5)	1.4 (1.1–1.8)	< 0.001	1.1 (0.8–1.5)	0.2
Performs sex under the influence of drugs		741 (45.5)	1345 (30.0)	1.9 (1.7–2.2)	< 0.001	1.9 (1.4–2.5)	< 0.001
Uses shared syringes		114 (10.0)	234 (7.0)	1.4 (1.1–1.8)	< 0.001		
Reported Previous STI diagnosis^a^		263 (16.0)	404 (9.0)	1.7 (1.4–2.0)	< 0.001	1.5 (1.09–2.2)	0.01
STI diagnosis at current visit^b^		85 (5.0)	208 (4.5)	1.1 (0.8–1.4)	0.3		

*OR* Odds Ratio, *CI* Confidence Interval, *STI* Sexually Transmitted Infection, *FSU* Former Soviet Union

^a^OR adjusted to age in the univariate analysis

^b^HIV, HBsAg, infectious syphilis, pharyngeal or urethral *N. Gonorrhea*, urethral *C. Trachomatis*

MPS who purchased sex often were more likely than MPS who purchased sex rarely or once to be older, married and foreign-born (Table 2). They were also more likely to engage in risky sexual behavior than MPS who purchased sex only once or rarely. This included having an early sexual debut, multiple sexual partners and performing sex under the influence of drugs.

**Table 2:** Univariate analysis of characteristics of MPS according to frequency of purchasing sex

		Once 363 (%)	Rarely 705 (%)	Often 391 (%)	*P*
Age	18–29	207 (53)	294 (39)	180 (36)	0.01
	≥30	183 (47)	4586 (61)	323 (64)	
Relationship status	Single (single, divorced & widowed)	300 (80)	515 (75)	199 (70)	< 0.001
	Married	76 (20)	171 (25)	84 (30)	
Country of birth	Israel	271 (70)	503 (67)	309 (62.5)	0.04
	Foreign-born	114 (30)	244 (33)	185 (37.5)	
Reason for attending clinic	Symptomatic or sex with a symptomatic partner	103 (26.5)	181 (24)	94 (18.5)	0.01
	Self-referral screening	288 (73.5)	572 (76)	411 (81.5)	
Age sexual debut < 18 years		185 (49)	371 (51)	318 (68.5)	< 0.001
≥ 11 sexual partners during lifetime		193 (51)	508 (69)	433 (91.5)	< 0.001
≥6 sexual partners within the last 3 months		14 (3.5)	39 (5.5)	250 (51.5)	< 0.001
Irregular or no use of condom during vaginal sex		298 (79)	579 (78)	357 (82)	0.3
Performs sex under the influence of drugs		145 (37.5)	323 (43.5)	273 (55.5)	< 0.001
Uses shared syringes		2 (1)	33 (6)	79 (22.5)	< 0.001
Reported Previous STI diagnosis		55 (14)	135 (18)	73 (14.5)	0.1
STI diagnosis at current visit^a^		13 (3.5)	40 (5.5)	32 (6.5)	0.1

^a^HIV, HBsAg, infectious syphilis, pharyngeal or urethral *N. Gonorrhea*, urethral *C. Trachomatis*

Men who were diagnosed with an STI during the clinic visit were more commonly non-Israeli born (mostly originated in the FSU and Africa) than the men who were not diagnosed with an STI (Table 3). In the univariate analysis, those who were STI positive were more likely to present with symptoms typically related to STI, engage in risky sexual behavior, used condoms inconsistently during vaginal sex, and were more likely to have non-Israeli born sexual encounters as compared to men who were STI-free in the current visit. In the multivariate analysis, being born in Africa, presenting with typical STI symptoms, reporting of a greater number of lifetime sexual partners and performing sex with a non-Israeli were significant determinants associated with an STI diagnosis at the current visit (Table 3). Purchasing sex was not associated with an STI diagnosis.

**Table 3:** Characteristics of heterosexual men visiting the clinic with at least one STI diagnosis^a^ vs. those without an STI diagnosis

		Univariate analysis				Multivariate analysis	
		Men diagnosed with at least one STI 293 (%)	Men without an STI diagnosis 5863 (%)	OR^c^ (95% CI)	*P*	OR (95% CI)	*P*
Age	18–29	152 (52.0)	2955 (50.5)	1 (ref)	0.3	1 (ref)	
	≥30	141 (48.0)	2883 (49.5)	0.8 (0.6–1.1)		0.7 (0.4–1.2)	0.2
Relationship status^b^	Single (single, divorced & widowed)	214 (82.0)	4146 (83.0)	1 (ref)	0.7		
	Married	47 (18.0)	866 (17.0)	1.0 (0.6–1.4)			
Country of birth	Israel	164 (56.5)	4047 (70.0)	1 (ref)		1 (ref)	
	Western Countries	9 (3.0)	215 (4.0)	0.8 (0.4–1.7)	0.7	1.0 (0.3–3.8)	0.8
	North Africa and Middle East	4 (1.5)	72 (1.0)	1.0 (0.3–3.0)	0.8	2.8 (0.5–14.1)	0.2
	The FSU	69 (24.0)	907 (16.0)	1.7 (1.2–2.2)	< 0.001	1.2 (0.5–2.4)	0.6
	Asia	8 (3.0)	84 (1.5)	1.6 (0.7–3.5)	0.1	2.1 (0.4–10.1)	0.3
	Africa	33 (11.0)	397 (7.0)	1.8 (1.2–2.7)	< 0.001	2.6 (1.2–5.5)	< 0.001
	South America	4 (1.5)	57 (1.0)	1.7 (0.6–4.8)	0.3	1.5 (0.1–13.6)	0.6
Reason for attending clinic	Symptomatic or sex with a symptomatic partner	129 (44.0)	1049 (18.0)	3.6 (2.8–4.5)	< 0.001	4.8 (3.0–7.8)	< 0.001
	Self-referral screening	164 (56.0)	4814 (82.0)	1 (ref)		1 (ref)	
Age sexual debut < 18 years		159 (57.0)	2900 (51.5)	1.3 (1.0–1.6)	0.02	1.2 (0.7–2.1)	0.3
≥ 11 sexual partners during lifetime ^b^		196 (69.0)	3260 (57.5)	1.7 (1.3–2.3)	< 0.001	3.6 (1.9–6.7)	< 0.001
≥6 sexual partners within the last 3 months		38 (13.5)	656 (12.0)	1.4 (0.9–2.0)	0.05		
Irregular or no use of condom during vaginal sex		239 (87.5)	4442 (80.5)	1.5 (1.1–2.3)	0.01	2.1 (0.9–5.1)	0.08
Irregular or no use of condom during anal sex		63 (75.0)	1344 (78.5)	0.8 (0.4–1.3)	0.4		
Purchased sex		85 (29.0)	1564 (26.5)	1.0 (0.7–1.3)	0.7	0.6 (0.3–1.0)	0.08
Performed sex with a non-Israeli partner within the last 3 months		34 (38.0)	407 (22.5)	2.0 (1.3–3.2)	< 0.001	1.7 (1.0–2.8)	0.02
Performs sex under the influence of drugs		115 (39.5)	1969 (34.0)	1.4 (1.1–1.8)	< 0.001	1.1 (0.6–2.0)	0.5
Uses shared syringes		12 (5.5)	336 (7.5)	1.0 (0.5–2.0)	0.7		
Circumcised		98 (78.0)	1460 (82.0)	0.7 (0.4–1.1)	0.1		
Reported Previous STI diagnosis^b^		48 (16.5)	619 (10.5)	1.2 (0.9–1.7)	0.1		

*OR* Odds Ratio, *CI* Confidence Interval, *STI* Sexually Transmitted Infection, *FSU* Former Soviet Union

^a^HIV, HBsAg, infectious syphilis, pharyngeal or urethral *N. Gonorrhea*, urethral *C. Trachomatis*

^b^OR adjusted to age in the univariate analysis

^c^adjusted to reason for attending clinic

## Discussion

### Characteristics of MPS

During the study period, 1649 men, who comprised of 26.7% (range 20.1–30.6%) of the heterosexual men who visited the clinic were MPS. However, our study included a captured audience, and refers to lifetime sex purchasing. This captured audience was treated in a special clinic, which is located centrally in Tel Aviv, in close proximity to an area highly populated with brothels. Its staff is trained to encourage open discussions regarding intimate sexual activity in a non-judgmental fashion. Men who consider themselves to be at a high risk for STI acquisition may feel more comfortable attending the clinic and reporting that they purchased sex. The estimates of MPS in this study is therefore higher than the general male population in Israel.

MPS in this study were more likely to be older, married, non-Israeli born, engage in additional sexual behaviors which are associated with risk for HIV/STI transmission and report previous STI diagnoses. Further, involvement in risky sexual behavior was correlated with the frequency of purchasing sex, i.e. the more often the MPS purchased sex the more likely he was to engage in risky sexual behavior.

Our finding that older age was a significant determinant of purchasing sex is similar to other studies [15, 16]. In a sample of men who were diagnosed with *N. Gonorrhea* in Tel Aviv, a greater percentage of men who purchased sex were 34 years of age or older compared to the men who did not purchase sex (45% vs. 33%, *p* < 0.03) [17]. This observation may reflect the increasing opportunities for purchasing sex during one’s lifetime. Additionally, older men more commonly have more financial resources to purchase sex than do younger men.

Married men were more likely to purchase sex than single men in this study, in parallel with other publications [16, 18]. Similarly, in Mor et al., a greater percentage of Israeli men who purchased sex were married compared to men who did not purchase sex (41% vs. 29%, *p* < 0.02) [17]. Married men who seek sexual encounters outside of their marriages may choose to purchase sex, as it may be more convenient for them rather than establishing a relationship with a non-paid casual sex partner. It is also possible that married MPS attended the clinic more so than married non-MPS due to a greater perception of risk of potentially infecting their wives. Furthermore, married men may attend the clinic more often due to the confidentiality afforded by the clinic, as the visit is not included in their regular medical record, which is kept by their personal doctor.

The observed higher proportion of MPS who were born in the FSU and Asia compared to the non-MPS may reflect a cultural difference in attitudes towards the practice of purchasing sex. In the FSU and Asian countries, FCSW may be considered more readily accessible and conspicuous [10, 19] than in Israel, possibly making the act of purchasing sex more socially acceptable. As reflected by the composition of the Israeli population, it is also reasonable to assume that the Asian and some FSU men attending the clinic were migrant workers. Several studies demonstrated that migrating men are a sub-population that purchases sex at a higher frequency than the local population [6, 20]. It has been suggested that migrants are driven to purchase sex due to loneliness and peer-pressure [20].

There was no statistical significant association between purchasing sex and STI burden despite the greater engagement of MPS in risky sexual behavior. This may be partially explained by the finding that MPS were more likely to use condoms regularly during vaginal sex than non-MPS. Other studies have also shown that MPS use condoms more regularly than non-MPS [15, 21]. It is possible that MPS were more aware of the risks pertaining to unprotected casual sex, or that MPS were more likely to be married than non-MPS and use condoms to protect them from possibly transmitting STI to their wives. The higher rate of condom use might be driven from the FCSW side, who may have encouraged condom use to protect their own health and to provide a physical barrier between themselves and their clients. Supporting this hypothesis, in Linhart 2008, most women in the sample of 300 FCSW in Tel Aviv reported regular condom use during vaginal sex (96.5%) [22]. Albeit, the riskier sexual profile of MPS demonstrated in this study indicates that in the case where an MPS does have an STI, he is at a greater likelihood to transmit it to other sexual partners than a non-MPS. This is further supported by an additional finding in our study showing that MPS had more previous STI diagnoses than non-MPS, in parallel with other studies [8, 16, 23].

### Health policy/sexual health promotion

The proposed new law criminalizing MPS is included in the National strategy to eliminate prostitution in Israel. According to the proposal, MPS who are condemned will be required to pay a fine or participate in an educational program, which emphasizes the negative moral and psychological consequences of commercial sex. As this study demonstrates that MPS engage in risky sexual behavior, we propose that this educational course utilize its captured audience and include a component on sexual health that promotes healthy sexual behaviors and sexual relationships as well as preventing STI.

According to the findings from this study, STI clinics can be used to reach MPS and potentially provide counselling workshops to MPS interested in altering their risky sexual behaviors. MPS who voluntarily want to undergo an educational course should be allowed to without having to face legal ramifications.

This first study in assessing sexual risk behaviors among MPS is subject to several limitations. First, a selection bias may exist as the male clinic attendants may belong a-priori to a high-risk population thus precluding making assumptions of the general male population. Second, there is potential recall and reporting bias, as sexual behaviors are associated with negative stigma. This under reporting bias, if exists, is probably similar among MPS and non-MPS, non-differential, and thus conservative. To minimize recall and reporting bias, men were questioned about their sexual behavior within the three months prior to their clinic’s visit and the questionnaire was completed in a non-judgmental and respectful approach by trained staff. Third, the cross-sectional study design limits the ability to draw any conclusions regarding a causal link between sex purchasing and STI prevalence. Finally, the time elapsed between purchasing sex and the clinic visit varies among clinic attendees.

## Conclusions

In summary, of the men who visited the clinic during 9 years of follow-up, 26.5% reported they had purchased sex. The riskier sexual profile of MPS reflects the potential of bridging STI to other low risk sexual networks. A recent governmental decision to adopt the Nordic model in criminalizing men who purchase sex is aiming to decrease sex purchasing and reduce the inextricable risk for STI transmission. This new legislation represents a shift in society’s perception of prostitution and targets MPS, as they provide the demand for prostitution. Taking advantage of this new proposed Israeli legislation, we suggest that STI prevention interventions targeted towards heterosexual MPS be included as a component of a larger, holistic approach that aims to eliminate sex purchasing by encouraging condom use and behavioral modification as well as psychosocial support and understanding the harmful impact of prostitution and its destructive impact on FCSW.

## Abbreviations

AIDS: Acquired immune deficiency syndrome
CI: Confidence interval
FCSW: Female commercial sex worker
FSU: Former Soviet Union
HIV: Human immunodeficiency virus
MPS: Men who pay for sex
OR: Odds Ratio
STI: Sexually transmitted infection
